# Visual disorders and driving ability in persons with dementia: A mini review

**DOI:** 10.3389/fnhum.2022.932820

**Published:** 2022-11-29

**Authors:** Eleni Papageorgiou, Daniil Tsirelis, Katerina Lazari, Vasileios Siokas, Efthimios Dardiotis, Evangelia E. Tsironi

**Affiliations:** ^1^Department of Ophthalmology, University Hospital of Larissa, Larissa, Greece; ^2^Department of Neurology, University Hospital of Larissa, University of Thessaly, Larissa, Greece; ^3^Department of Ophthalmology, School of Health Sciences, Faculty of Medicine, University Hospital of Larissa, University of Thessaly, Larissa, Greece

**Keywords:** driving, dementia, Alzheimer’s disease (AD), cognitive impairment (CI), visual impairment, retinal imaging

## Abstract

**Background:**

Impaired driving ability in patients with Alzheimer’s disease (AD) is associated with a decline in cognitive processes and a deterioration of their basic sensory visual functions. Although a variety of ocular abnormalities have been described in patients with AD, little is known about the impact of those visual disorders on their driving performance.

**Aim:**

Aim of this mini-review is to provide an update on the driving ability of patients with dementia and summarize the primary visual disorders affecting their driving behavior.

**Methods:**

Databases were screened for studies investigating dementia, associated visual abnormalities and driving ability.

**Results:**

There is consistent evidence that dementia affects driving ability. Patients with dementia present with a variety of visual disorders, such as visual acuity reduction, visual field defects, impaired contrast sensitivity, decline in color vision and age-related pathological changes, that may have a negative impact on their driving ability. However, there is a paucity in studies describing the impact of oculovisual decline on the driving ability of AD subjects. A bidirectional association between cognitive and visual impairment (VI) has been described.

**Conclusion:**

Given the bidirectional association between VI and dementia, vision screening and cognitive assessment of the older driver should aim to identify at-risk individuals and employ timely strategies for treatment of both cognitive and ocular problems. Future studies should characterize the basic visual sensory status of AD patients participating in driving studies, and investigate the impact of vision abnormalities on their driving performance.

## Introduction

Dementia is characterized by deterioration in cognitive function beyond what might be expected from the usual consequences of biological aging (World Health Organization [WHO], 2021). Currently more than 55 million people worldwide have been diagnosed with dementia, with Alzheimer’s disease (AD) being the most common form of dementia (World Health Organization [WHO], 2021). Driving is a highly complicated task that requires multiple cognitive functions, such as attention, visuoperceptual and visuospatial abilities, which may be considerably compromised in individuals with dementia (Reger et al., 2004; Hird et al., 2016).

Intact vision is one of the essential requirements for safe driving, as more than 90% of the sensory information during driving is considered to be visual. Recent evidence has suggested increased motor vehicle crash risk with binocular visual field impairment (Kasneci et al., 2014; Wood et al., 2022). Visual acuity (VA) has been weakly related to crash involvement (Owsley and McGwin, 1999) and there is no evidence of increased crash risk in drivers with mild VA impairment (Wood et al., 2022). As the retina is a developmental outgrowth of the diencephalon, it is also affected by neurodegeneration, and dementia has been associated with a variety of visual disorders from the retina to the visual cortex. Those alterations could have a negative impact on driving ability (Katz and Rimmer, 1989; Sadun and Bassi, 1990) and visual impairment (VI) has been considered one of the early symptoms of dementia (Kusne et al., 2017). Visual abnormalities, such as reduced contrast sensitivity, visual field defects and loss of color vision have been often described in AD patients (Katz and Rimmer, 1989). Additionally, recent advances of *in vivo* ocular imaging have also revealed retinal and optic nerve alterations in AD patients, such as retinal ganglion cell (RGC) loss, nerve fiber layer atrophy, retinal thinning, amyloid β-protein (Aβ) accumulation, and amyloid-related neurodegeneration in the retina (Curcio and Drucker, 1993; Berisha et al., 2007; Perrin et al., 2009; Guo et al., 2010; Jindahra et al., 2010).

As the world population is aging quickly and one in three dementia patients still drives (Foley et al., 2000; Silverstein, 2008), it is crucial to understand the primary visual disorders affecting the driving ability of AD patients, in order to ensure on-road safety without violating individual autonomy for fit-to-drive individuals. Although there are several reports on driving with dementia, studies assessing the driving ability of persons with dementia in regard to their VI are scarce. The objective of this mini-review is to provide an update on the driving ability of individuals with AD and summarize the visual disorders affecting their driving behavior.

A PubMed search of articles published from January 2000 to April 2022 on the driving ability and oculo-visual disorders of persons with AD was performed (human and English language only). Selected key articles published before 2000 were also included. Searches included a combination of the following terms: cognitive impairment, dementia, Alzheimer’s disease, driving, on-road test, driving simulator, neuropsychological tests, traffic accidents, visual acuity, visual search, retinal imaging, and visual field. The references were then reviewed for pertinent articles, resulting in 20 eligible studies (Table 1).

**TABLE 1:** Baseline characteristics of studies examining driving aspects in patients with cognitive decline and dementia.

References	Study participants	Type of driving evaluation	Mean age of participants	Results
Fitten et al., 1995	13 AD 12 VaD 15 Diabetes mellitus 26 healthy older controls 16 young controls **Corrected VA of 20/40 or better**	On-road driving test	Mean age dementia: 70.8 Mean age young controls: 27.6 Mean age older controls: 71.8	The drive scores of AD and VaD were significantly worse from the 3 control groups and MMSE was the best predictor of driving ability
Dobbs, 1997	115 dementia (vast majority AD) 35 older controls 23 young controls	On-road test, cognitive and motor tests predictive of driving performance	Mean age dementia: 72 mean age older controls: 69 mean age young controls: 36	MMSE can be useful for driving evaluation, 25% of patients with dementia showed safe driving behavior
Rizzo et al., 1997	21 AD 18 controls **No difference in VA between groups**	Driving simulator	Mean age dementia: 71.5 Mean age controls: 71.9	29% of patients with AD experienced crashes vs. 0 of controls, strong predictors of crashes included visuospatial impairment, reduction in the useful field of view (UFOV), and reduced perception of 3-dimensional structure-from-motion
Carr et al., 2000	63 AD (very mild/mild) 58 controls **Subjects with significant visual decline excluded**	Questionnaire	Mean age AD: 77	AD participants drive less than controls, but with the same crash rate
Rizzo et al., 2001	18 patients with AD 12 controls **No difference in VA between groups**	Driving simulator	Mean age dementia: 73 Mean age controls: 70	Higher simulator crash rate in patients with AD (*p* < 0.05%), predictors of crashes included visuospatial impairment, disordered attention, reduced processing of visual motion cues and overall cognitive decline, the AD group had worse UFOV scores
Ott et al., 2003	PART A: 18 very mild AD 9 mild AD PART B: 6 normals 21 probable AD 11 MCI 1 FTD 1 mixed dementia **No subjects had visual impairment (VI) that would impair their driving ability**	Caregiver-reported driving ability	PART A: Mean age: 74.8 (48.1% male) PART B: Mean age: 73.8 (47.5% male)	Higher frequency of crashes in patients with dementia Computerized tests of maze performance identified driving impairment
Duchek et al., 2003	29 mild AD 21 very mild AD 58 controls **Participants had a valid driver’s license and *VA* > 20/50**	Standardized on-road test over repeated times of testing	Mean age mild AD: 74.2 (52% male) Mean age very mild AD: 73.7 (76% male) Mean age controls: 77 (52% male)	Decline in driving performance over time, primarily in early-stage AD
Uc et al., 2004	32 probable/mild AD 136 elderly controls **Participants had a valid driver’s license** **Patients had worse near and far VA, contrast sensitivity and structure from motion**	Visual and cognitive tests On-road (route-finding) test	Mean age dementia: 75.9 Mean age controls: 64	AD patients made more incorrect turns, got lost more often, and made more at-fault safety errors, which were predicted using scores of visual and cognitive tests
Uc et al., 2005	33 patients with mild AD 137 controls **Participants had a valid driver’s license Patients had worse near and far VA, contrast sensitivity and structure from motion**	Visual and cognitive tests On-road performance	Mean age dementia: 76.1 (84.8% male) Mean age controls: 64.3 (50.3% male)	Worse performance in patients with AD, impaired visual search and recognition of traffic signs
Brown et al., 2005	17 mild AD 33 very mild AD 25 elderly controls **Corrected visual acuity was better than 20/50 and visual fields were normal on confrontation testing**	On-road test: WURT	Mean age mild AD: 73.2 (58.8% male) Mean age very mild AD: 77.1 (63.6% male) Mean age controls: 72.4 (40% male)	The control group performed significantly better on the road test than the AD groups
Dawson et al., 2009	40 patients with mild AD 115 controls **Subjects with AD were older and had worse near and far VA, contrast sensitivity, structure from motion and had more vision-related comorbidities, but this was not significant**	Naturalistic on-road driving test	Mean age AD: 75.1 (82.5% male) Mean age controls: 69.4 (52.2% male)	Patients with AD had significantly more safety errors than controls and the most common errors were lane violations
Frittelli et al., 2009	20 patients with mild AD 20 patients with MCI 19 controls **Participants had a valid driver’s license and *VA* > 20/50**	Driving simulator	Mean age AD: 72 Mean age MCI: 71.6 Mean age controls: 68.9	Drivers with AD were rated as significantly worse than MCI subjects and healthy elderly drivers on several driving behaviors
Seiler et al., 2012	194 AD, 12 VaD, 11 DLB, 16 FTD, 7 other type	Questionnaires	Mean age dementia: 74.2 (60.4% male)	Highest rate of driving cessation on DLB (>90%)
Davis et al., 2012	59 patients with AD 44 controls **Participants underwent a vision screen**	Naturalistic on-road driving test (RIRT)	Mean age dementia: 76 (49.2% male) Mean age controls: 71.2 (38.6% male)	Patients with AD scored worse on both naturalistic and on-road driving
Eby et al., 2012	17 patients with mild AD 26 controls	Naturalistic on-road driving test	Mean age dementia: 64.5 (76% male) Mean age controls: 50% (50% male)	Patents with dementia drove less miles, drove slower, less destinations and more during the day
Etienne et al., 2013	10 early AD 29 controls **Participants had *VA* > 20/40**	Neuropsychological tests and static driving simulator (IFSTTAR)	Mean age dementia: 74.68 Mean age controls: 70.83	AD patients have slower reaction times on neuropsychological tests
Mauri et al., 2014	106 AD, 37 AD with vascular signs, 30 DLB and Parkinsonisms, 18 VaD, 7 FTD **6% of patients had reduction of VA not severe enough to compromise their ability to drive**	Interview gathering information about driving ability	Mean age dementia: 73.8 (71.7% male)	Lower MMSE scores correlated with self-limitation of driving
Vella and Lincoln, 2014	18 AD 4 VaD 2 unknown subtype **Patients were able to read 12 point text**	Nottingham Neurological Driving Assessment	Mean age dementia: 73 (75% male)	Rookwood Driving Battery (RDB) is stricter than Dementia Drivers’ Screening Assessment (DDSA) when indicating driving cessation
Aksan et al., 2015	32 patients (probable) AD 32 patient PD 77 controls **Controls performed better than patients in visual sensory functioning (near and far visual acuity and contrast sensitivity)**	Naturalistic on-road driving test	Mean age dementia: 77.6 Mean age controls: 75.4	On-road impairment (navigation-related secondary task performance)
Fuermaier et al., 2019	80 AD, 59 VaD FTD DLB PD 45 controls **Patients had minimum VA of 20/40 and a minimum horizontal field of view of 120 degrees**	Test ride investigating practical fitness to drive (TRIP)	Mean age dementia: 71.7 Mean age controls: 76.3	On-road fails associated with clinical, neuropsychological and driving simulator assessments on AD participants. VaD higher rate of driving cessation compared to mixed AD and Vascular signs

AD, Alzheimer’s disease; DLB, dementia with Lewy bodies; VaD, vascular dementia; VA, visual acuity; FTD, frontotemporal dementia; UFOV, useful field of view test; MCI, mild cognitive impairment; PD, Parkinson’s disease; MMSE, mini mental state exam; IFSTTAR, french institute of science and technology for transport, development and networks; WURT, washington university road test; RIRT, rhode island rhode test.

## Driving ability and oculo-visual disorders in dementia

### The role of vision in driving

Driving is inevitably a complex visuoperceptual and motor task, which is largely dependent on intact acquisition and processing of visual information. Numerous studies have attempted to define the impact of vision disorders on driving ability by assessing visual measures, such as visual field, VA, contrast sensitivity, glare sensitivity, and useful field of view (UFOV). However, the minimum requirements for the visual function of drivers are highly variable across countries and research is still ongoing. Most studies have identified a weak or no relationship between VA and increased crash risk, however, VA is included as a general measure of vision in the licensing standards of most countries (Owsley and McGwin, 1999; Wood, 2019). A recent systematic review reported no evidence of increased crash risk with mild VA impairment, but increased crash risk with binocular visual field impairment (Wood et al., 2022). The minimum binocular field in Europe should be at least 120° in the horizontal meridian and approximately evenly divided to the left and right of fixation (EU Working Group, 2009). Additionally, no significant defects should be within 20° above and below fixation (EU Working Group, 2009). The UFOV test additionally assesses visual attention and visual processing speed and has been related to crash involvement in older adults (Sekuler and Ball, 1986; Owsley and McGwin, 1999). Impairment of contrast sensitivity has been also linked to deterioration of driving performance in some studies (Owsley and McGwin, 1999). Specific eye conditions of the elderly, who may suffer from dementia as well, have a negative impact on driving performance and safety. Cataract compromises VA, visual field, contrast sensitivity, increases glare, and higher crash risk has been reported in older adults with cataracts (Owsley and McGwin, 1999; Wood and Carberry, 2004; Swan et al., 2019; Wood, 2019). Age-related macular degeneration (AMD) causes deterioration of VA and central field loss and may be associated with impaired driving ability (Wood, 2019). Drivers with visual field loss from glaucoma also report several driving difficulties and display higher collision risk (Wood, 2019). Finally, individuals with homonymous hemianopia are prohibited from driving in many countries, however, some of them may exhibit safe driving skills by means of compensatory eye and head movements (Owsley and McGwin, 1999; Papageorgiou et al., 2012; Kasneci et al., 2014; Bowers, 2016). Although a systematic review showed mixed evidence regarding the impact of cataract, glaucoma, age-related macular degeneration, and homonymous field loss on motor vehicle collision risk, it is clear that the presence of those conditions in older, at-risk for dementia adults deserves careful consideration regarding their fitness to drive (Wood et al., 2022).

### Studies on the driving ability of patients with dementia

A summary of the studies assessing the driving ability of patients with AD is presented in chronological order in Table 1. Eleven studies used on-road driving tests, four studies used a driving simulator and five studies used questionnaires and interviews for assessment of driving ability.

#### Alzheimer’s disease

Alzheimer’s disease (AD) is the most common type of dementia (Nichols et al., 2022). It is a major socio-economic issue, and it is expected that that the total number of people suffering from AD in 2050 will be 13.8 million (Hebert et al., 2013). Several risk factors seem to influence the incidence of AD, with age being the most important (Guerreiro and Bras, 2015). Clinically, AD is mainly characterized by deficits in memory, language, executive, and visuospatial domains (Dubois et al., 2021; Graff-Radford et al., 2021). The impairment of those cognitive and visuospatial functions affects the driving ability of people with dementia and it has been reported that drivers with AD are 2.5–4.7 times more likely than age-matched controls to be involved in vehicle collisions (Friedland et al., 1988; Tuokko et al., 1995). Additionally, the majority of patients with AD voluntarily cease or limit driving (Carr and Ott, 2010) and some of them adjust their driving habits, i.e., they drive fewer miles per year, avoid driving in unfamiliar situations, and drive with another person (Davis et al., 2012; Feng et al., 2020; Davis and Owens, 2021). On the other hand, many drivers with mild dementia are able to pass an on-road driving test (Duchek et al., 2003; Brown et al., 2005; Ott et al., 2008; Iverson et al., 2010).

Studies on the driving ability of persons with AD are heterogenous in terms of the tools used for driving assessment and do not always have consistent results. Most authors have concluded that neuropsychological batteries, such as the Mini-Mental State Examination (MMSE), the Rookwood Driving Battery (RDB), and the Dementia Drivers’ Screening Assessment (DDSA), can be used as indicators for driving ability in conjunction with simulator and on-road tests (Dobbs, 1997; Mauri et al., 2014; Vella and Lincoln, 2014).

Most naturalistic and standardized on-road tests suggest that AD patients perform more driving safety errors and their driving ability is inferior to that of healthy elderly individuals (Zuin et al., 2002; Uc et al., 2004; Lincoln et al., 2006; Dawson et al., 2009; Aksan et al., 2015; Barco et al., 2015). Recently, Davis et al. (2020) showed that drivers with dementia undergoing a license review were far more likely to have their license denied than those without dementia (69.2% vs. 10.3%). Fuermaier et al. (2019) found that individuals with AD, who failed the on-road test, had worse results on operational, tactical and visual aspects of driving compared to individuals with AD who passed the on-road test. In a study by Uc et al. (2005), AD patients reported a significantly lower number of both landmarks and traffic signs, and visual and cognitive screening tests were able to predict the impairment of visual search and identification of roadside landmarks.

Driving simulators have been an effective alternative to the on-road tests for assessing fitness to drive in patients with neurological conditions, due to their safety profile, use of standardized interfaces, and availability of various repeatable scenarios (Etienne et al., 2013). A preliminary study in the Iowa driving stimulator found that 29% of AD patients experienced a crash versus none in the control group (Rizzo et al., 1997). Static spatial contrast sensitivity and UFOV were compromised in AD patients and visual and cognitive evaluations were able to predict patients who would have a crash (Rizzo et al., 1997). Interestingly, some patients of the AD group who experienced a crash, were looking directly at the road but did not avoid the crash. A possible explanation was the “looking without seeing” attitude, which has been described in patients with dorsolateral lesions of the visual cortex due to stroke or AD (Rizzo and Hurtig, 1987; Hof et al., 1990; Mendez et al., 1990; Rizzo, 1993; Davis et al., 2020).

Most studies agree that persons with dementia are more likely to fail a road test than healthy controls and that even mild dementia is associated with impaired driving abilities and a substantially higher risk of failing road tests (Chee et al., 2017). In order to ensure traffic safety, a large body of literature has focused on the understanding of factors associated with impaired driving ability of AD patients and the development of appropriate test batteries to identify unsafe AD drivers. Cognitive processes related to driving errors in AD include attention, memory, decision making, processing speed, executive functioning, and visuospatial orientation (Estevez-Gonzalez et al., 2003; Uc et al., 2005; Ott and Daiello, 2010). However, a variety of sensory system disorders, such as VI and auditory dysfunction, may also negatively affect the driving performance of AD patients. Although the visual manifestations of AD have been extensively described, little is known about the visual deficits associated with impaired driving performance of AD patients.

#### Assessment of basic visual sensory functions in driving studies with Alzheimer’s disease patients

The cited studies have focused on the decline of cognitive skills and the investigation of neuropsychological tests assessing primarily visuoperceptual, attentional, and executive functions. Seventeen of 20 studies have included age-matched control subjects. Interestingly, there is a paucity in publications assessing the effect of oculovisual decline on the driving ability of AD subjects. Most authors have matched the visual status of patients and controls, but no detailed information is given regarding the ophthalmological tests used. Seventeen of 20 studies have included vision testing in their eligibility criteria for AD patients and controls. Seven studies required a minimum VA of 20/40 or 20/50 sufficient for driving according to current legislations, two studies have included AD patients and controls with equal VA (Rizzo et al., 1997, 2001), and one study reported that participants underwent a vision screen. Four studies did not report on the VA of participants, but included a statement that subjects with significant visual decline were excluded or they had a valid driver’s license (Carr et al., 2000; Ott et al., 2003; Uc et al., 2004, 2005). In the study by Brown et al. (2005) normal confrontation visual fields were required for study participants and Fuermaier et al. (2019) required a minimum horizontal field of view of 120 degrees. However, in almost all studies no additional basic visual sensory function tests were reported and no specific information about the co-existence of age-related eye conditions in the study population, such as cataracts or AMD, was given. Although the above studies included participants with no VI that would impair their driving ability, further data analysis by some authors showed that patients had worse near and far VA, contrast sensitivity and structure from motion (Rizzo et al., 1997, 2001; Uc et al., 2004, 2005; Dawson et al., 2009; Aksan et al., 2015). Dawson et al. (2009) found that patients had more vision-related comorbidities, but this was not significant. Patients with significant motor impairment have been excluded from the cited studies.

In conclusion, although intact vision plays an important role in driving and AD patients may have ocular pathologies related to dementia and age, basic visual sensory function in driving studies with AD patients has received little attention.

### Ocular manifestations in patients with dementia

Current literature suggests that there are several ophthalmological manifestations of AD, which may be relevant for driving performance. Oculovisual alterations in AD patients are due to primary involvement of the visual pathway and higher cortical impairment. Changes in primary vision include reduction of visual acuity, impaired color vision, loss of contrast sensitivity, visual field defects, decreased stereopsis, defective smooth pursuit, and saccadic eye movements (Table 2; Cormack et al., 2000; Pache et al., 2003; Risacher et al., 2013; Nolan et al., 2014; Kim et al., 2022). Higher order visual functions have also been reported to affect visuospatial function, eye-head coordination, motion detection, and identification of objects (Table 2). This review focuses on the disorders of primary vision, which may affect the driving potential of AD patients.

**TABLE 2:** Visual function and ocular structure alterations in dementia.

Visual function	Findings in AD
Visual acuity	Impaired in advanced stages of AD (Friedman et al., 2002)
Color vision	Compromised color discrimination (Pache et al., 2003)
Contrast sensitivity	Reduced (Cormack et al., 2000)
Visual field	Impaired, inferior hemifield loss (Trick et al., 1995)
Motion perception	Higher threshold for detecting moving objects (Gilmore et al., 1994)
Stereopsis	Compromised (Thiyagesh et al., 2009)
Eye movements	Increased saccadic latency and hypometric saccades (Yang et al., 2013)
Pupillary function	Atypical response to mydriatics, impaired amplitude and latency of light reflex and larger pupil size (Tales et al., 2001)
ERG	Abnormal pattern ERG (Katz et al., 1989; Trick et al., 1989)
VEP	Abnormal pattern VEP and reduction of P1 and N1 VEP amplitudes (Stothart et al., 2015)
**Ocular structures**	
Lens	Aβ deposits in lens (supranuclearly) and aqueous humor (Goldstein et al., 2003)
Retina	Aβ and Tau deposits, retinal thinning, RNFL thinning, reduction of RGC axons (peripapillary and macular), enlargement of foveal avascular zone, compromised retinal blood flow (Liu et al., 2009; Kesler et al., 2011; Williams et al., 2015; Koronyo et al., 2017)
Choroid	Thinning of choroid (Gharbiya et al., 2014)
Optic nerve	Larger cup-to-disk ratio and pallor (Tsai et al., 1991; Lu et al., 2010)

AD, Alzheimer’s disease; ERG, electroretinogram; VEP, visual evoked potential; RNFL, retinal nerve fiber layer; RGC, retinal ganglion cell.

Pathological changes have been described in the crystalline lens, retina, optic nerve, and visual cortex of patients with AD (Table 2). Aβ deposits have been detected in the crystalline lens and aqueous humor, and a specific supranuclear cataract has been described in AD patients (Goldstein et al., 2003). The retina in AD may undergo similar pathological changes to the brain and recent research has focused in detecting retinal biomarkers for AD. Many authors have found Aβ deposits and pTau plaques in AD retinas (Koronyo-Hamaoui et al., 2011; Koronyo et al., 2012; Frost et al., 2014; Williams et al., 2015; La Morgia et al., 2016), although their presence has not been consistent across studies (Schön et al., 2012; Ho et al., 2014).

Recently, optical coherence tomography (OCT) has revealed a reduction in RGCs, retinal nerve fiber layer (RNFL) thickness and macular thickness in AD (Parisi et al., 2001; Paquet et al., 2007; Kesler et al., 2011; Kirbas et al., 2013; Chan et al., 2019). A recent meta-analysis of 11 OCT studies in AD showed that mean RNFL is significantly reduced in all four retinal quadrants around the macula (Coppola et al., 2015). Interestingly, RNFL changes have been demonstrated in early AD patients without VI, suggesting the potential use of RNFL as an early diagnostic marker (Lu et al., 2010; Bambo et al., 2014; Garcia-Martin et al., 2014). As AD progresses, a further decrease in RNFL thickness has been observed, and there was a significant correlation between overall macular volume and severity of cognitive impairment measured by the MMSE (Iseri et al., 2006; Tas et al., 2015). Specific to the optic nerve, imaging studies have demonstrated *in vivo* evidence of optic nerve head pathology in AD patients, such as larger cup-to-disk ratio and increased pallor of the optic nerve (Tsai et al., 1991; Lu et al., 2010; Bambo et al., 2015), reflecting a significant loss of RGC axons and/or possible retrograde degeneration affecting the retina (Danesh-Meyer et al., 2006). It has been shown that patients with AD have visual dysfunction, such as impairment of color vision and contrast sensitivity, that correlate with structural OCT changes, namely, RNFL thinning and macular thinning (Polo et al., 2017). Moreover, retinal vascular abnormalities, such as reduction in retinal microvasculature, narrowing of retinal veins (Cheung et al., 2014; Williams et al., 2015), reduced retinal blood flow (Feke et al., 2015), increased tortuosity, compromised branching complexity, and reduced choroidal thickness (Gharbiya et al., 2014; Bayhan et al., 2015) have been described.

Visual field defects in patients with AD are of special interest, because a minimum extent of the visual field is currently required from driving license authorities all over the world. In their preliminary study, Trick et al. (1995) showed that visual field loss in AD is most pronounced in the inferonasal and inferotemporal visual field but also involves the central region. Interestingly, the degree of loss correlated with the degree of dementia (Armstrong, 1996), and the predominant inferior field defects were attributed to pathological differences between cuneal and lingual gyri (Armstrong, 1996). Studies with frequency doubling technology (FDT) perimetry have described significantly greater false-negatives, test duration, and abnormal visual fields in AD patients compared with controls, with five-times greater (27.5%) frequency of glaucoma-like alterations in visual fields in AD (Aykan et al., 2013; Cesareo et al., 2015).

### Bidirectional association between visual impairment and dementia

Apart from the specific oculo-visual changes associated with dementia, older individuals may also experience age-related visual disorders, such as AMD, glaucoma, and cataracts. Such conditions may further impair the driving ability of affected patients and pose a risk for public safety or lead to driving cessation and loss of personal autonomy (Wood et al., 2018). Interestingly, a number of studies have linked many age-related eye diseases to cognitive impairment and dementia (Chung et al., 2015; Gupta et al., 2019; Rong et al., 2019; Zhang et al., 2019; Belamkar et al., 2021). An increased occurrence of AMD has been found in patients with AD (Klaver et al., 1999; Nolan et al., 2014) and Ab has been also identified in retinal drusen, which are a hallmark of AMD (Ratnayaka et al., 2015). An association of glaucoma and AD is also under investigation (Ramirez et al., 2017; Zhao et al., 2021). Two longitudinal studies have shown that poorer visual acuity was often a predictor of higher dementia incidence over time (Lee et al., 2020; Tran et al., 2020). Chen et al. (2021) found a bidirectional association between VI and dementia. In a retrospective study of 10,676 individuals over 65 years, they reported that patients who present with VI at the first examination are more probable to develop dementia in the future. On the other hand, patients with dementia are at a greater risk to experience visual loss over time (Chen et al., 2021). A recent meta-analysis has also revealed a bidirectional relationship between VI and cognitive impairment, suggesting that VI is a risk factor of cognitive impairment and individuals with cognitive impairment were more likely to have VI, although more evidence is needed to confirm the latter finding (Vu et al., 2021). Another systematic meta-analysis concluded that VI is related to an increased risk of both dementia and cognitive impairment in older adults, hence screening and treating vision impairment may help to alleviate the global burden of dementia (Shang et al., 2021). Possible mechanisms for the association of low vision and impaired cognition include loss of visual sensory information with resulting neuronal atrophy, errors in perceptual processing and consequent decline in higher-order cognitive performance, restricted participation in mentally stimulating activities due to visual loss and other common risk factors for both dementia and VI, such as vascular disease (Chen et al., 2021; Shang et al., 2021; Vu et al., 2021).

## Discussion

To date, several studies have examined the driving ability of patients with AD, and although there is significant methodological heterogeneity, it is clear that AD negatively affects driving ability. However, there is still no consensus regarding the cognitive and driving tests that have the best predictive value for fitness-to-drive in patients. This review highlights the need to characterize in more detail the basic visual sensory status of AD patients participating in driving studies, and investigate the impact of vision abnormalities on their driving performance. Research has shown that visual parameters substantial for safe driving, such as visual acuity, visual field, contrast sensitivity, and color vision, may be impaired in AD, and this field deserves further investigation. Larger studies should aim to compare AD patients with intact vision, AD patients with ocular disease, and age-matched controls, in order to assess the relative contributions of primary vision and cognitive impairment on their driving ability.

Additionally, current evidence on the link between visual disorders and dementia suggests the need for early screening and detection of older adults with VI. The bidirectional association between VI and dementia warrants both the vision examination of cognitively impaired individuals and also the cognitive assessment of older adults with visual disorders (Chen et al., 2021; Shang et al., 2021; Vu et al., 2021). Vision screening and cognitive assessment of the older driver should aim to identify at-risk individuals and employ timely strategies for treatment of both cognitive and ocular problems. A multidisciplinary team approach by eye care professionals, neurologists, geriatricians, and primary care providers could play an important role in helping affected patients to preserve mobility and independence for as long as possible after the onset of dementia and at the same time minimize individual clinical and public health consequences.

The importance of the recent advances in ocular imaging of dementia patients lies in the early identification of *in vivo* retinal and optic nerve changes, which may underlie driving-specific VIs and may have a future role as predictors of disease severity and fitness-to-drive. The impact of these retinal alterations on visual acuity and visual function of AD patients, especially in terms of their driving ability, still remains to be elucidated. However, these research findings suggest that retinal imaging is a promising tool for assessment of at-risk individuals, and may prove useful in the future for assisting physicians to decisions regarding fitness-to-drive.

## Author contributions

EP, DT, VS, and KL have been involved in drafting the manuscript or revising it critically for important intellectual content. ED and ET have given final approval of the version to be published. All authors made substantial contributions to conception and design, acquisition of data, and analysis and interpretation of data.
